# 肺大细胞神经内分泌癌分子标志物的研究进展

**DOI:** 10.3779/j.issn.1009-3419.2020.101.46

**Published:** 2020-11-20

**Authors:** 瑾瑶 张, 琳 杨, 峻岭 李

**Affiliations:** 1 100021 北京，国家癌症中心/国家肿瘤临床医学研究中心/中国医学科学院北京协和医学院肿瘤医院肿瘤内科 Department of Medical Oncology, National Cancer Center/National Clinical Research Center/Cancer Hospital, Chinese Academy of Medical Sciences and Peking Union Medical College, Beijing 100021, China; 2 100021 北京，国家癌症中心/国家肿瘤临床医学研究中心/中国医学科学院北京协和医学院肿瘤医院病理科 Department of Pathology, National Cancer Center/National Clinical Research Center/Cancer Hospital, Chinese Academy of Medical Sciences and Peking Union Medical College, Beijing 100021, China

**Keywords:** 肺大细胞神经内分泌癌, 分子分型, 诊断相关标志物, 预后相关标志物, Pulmonary large cell neuroendocrine carcinoma, Molecular subtype, Diagnostic-related marker, Prognostic-related marker

## Abstract

肺大细胞神经内分泌癌(pulmonary large cell neuroendocrine carcinoma, LCNEC)为肺神经内分泌癌的一种病理亚型，其发病率占肺癌手术标本的2.4%-3.1%，具有侵袭性高、预后差的特点，与吸烟高度相关。因发病率低、样本量少的原因导致相关研究较少，因此在临床上对肺LCNEC的诊断及治疗相对困难。本文结合近年肺大细胞神经内分泌癌相关基因测序及分子标志物的研究进展，对其分子分型、诊断及预后相关生物标志物进行阐述，为下一步研究方案寻找方向。

## Abstract (en)

Pulmonary large cell neuroendocrine carcinoma (LCNEC) is a pathological subtype of lung neuroendocrine cancer, which accounts for 2.4%-3.1% in surgical specimens of lung cancer. It is characterized by high invasiveness and poor prognosis, and highly correlated with smoking. There are few relevant studies due to the low incidence and small sample size. Therefore, it is relatively difficult to diagnosis and treatment in clinical practice. In this review, we described molecular subtype, diagnostic and prognostic-related markers about large cell neuroendocrine carcinoma of lung based on the recent progress in genomic sequencing and molecular markers, to find the direction for the next research.

目前，肺神经内分泌肿瘤(neuroendocrine tumor, NET)分为4种亚型：典型类癌(typical carcinoid, TC)、非典型类癌(atypical carcinoid, AC)、小细胞肺癌(small cell lung cancer, SCLC)和大细胞神经内分泌癌(large cell neuroendocrine carcinoma, LCNEC)^[1]^。其中，肺LCNEC的发病率占肺癌手术标本的2.4%-3.1%^[1-3]^，属于高级别神经内分泌癌(high grade neuroendocrine carcinoma, HGNEC)，具有侵袭性高、预后差的特点; 患者发病的平均年龄在65岁左右，且与吸烟高度相关^[4, 5]^。大部分患者确诊时疾病已处于中晚期，手术治疗效果有限，术后复发几率大，需要结合全身治疗来提高疗效^[5]^;因此，需对肺LCNEC进行探索，寻找有效的治疗靶点及筛选治疗敏感的患者。但因发病率低、样本量少的原因，有关肺LCNEC的分子分型、分子诊断及预后分子标志物的相关研究较少，仅近年来有所进展。本文结合近几年国内外有关肺LCNEC的基因分子研究进行阐述。

## 1 肺大细胞神经内分泌癌的基因图谱改变

### 1.1 基因突变

研究^[6-8]^表明在肺LCNEC中，大部分突变的基因是抑癌基因，且与吸烟相关，其基因图谱的改变与SCLC相似。George等^[8]^对60例LCNEC组织进行全基因组测序和69例进行转录组测序; 与正常组织相比，发现具有意义的基因突变分别是*TP53*、*RB1*、*STK11*、*KEAP1*及*RAS*(*KRAS/NRAS/HRAS*)通路上的基因; 在肺LCNEC中出现的比例分别是92%、42%、30%、22%和10%。在40%LCNEC病例中发现了*TP53*和*RB1*这两个基因的双等位基因改变; 值得注意的是，与其他组织学成分混合的LCNEC病例大多具有*RB1*的突变^[8]^。这些基因的突变类型主要是点突变和缺失突变^[8]^。另有学者^[9]^对32例肺LCNEC组织进行测序，同样也发现了*TP53*、*STK11*、*PTEN*等基因的改变。同时，也证实了在肺LCNEC中*NOTCH1-4*基因的突变率比较高，并引起NOTCH通路的改变，最终影响神经内分泌的分化^[7, 10-12]^。共同参与神经内分泌分化的基因还有*YAP1*、*hASH1*、*Hes1*、*Bcl-2*、*DLL1*和*NeuroD*等，而这些基因则是通过调节表达量来参与神经内分泌分化^[8, 13-15]^。和肺类癌一样，染色质重塑相关的基因也发生突变; 例如：*MEN1*、*ARID1A*、*ARID1B*及*MLL1-3*^[7, 11, 12]^。在PI3K-AKT-mTOR通路上也发现了相关基因的突变; 其中，*PTEN*突变率在肺LCNEC中占7%，PI3KCA占11%^[7, 8, 12]^。其他研究也证实了在肺LCNEC中PI3K-AKT-mTOR通路上的*PIK3CA*、*PTEN*、*AKT*、*RICTOR*和*mTOR*均发生突变，其突变概率分别为3%、4%、24%、5%及1%^[11]^。

### 1.2 基因扩增

在肺LCNEC中不仅有基因突变，还存在大量基因的扩增。George等^[8]^对肺*LCNEC*基因测序数据进行分析时发现了一些扩增比例较高的基因，分别是*MYC*、*MYCL1*、*NKX2-1*、*FGFR1*和*IRS2*。*MYCN*、*SOX2*及*CCEN1*在肺LCNEC中扩增比例也较高，比例分别为2%、11%和9%^[7]^。

### 1.3 特异基因改变

与其他肺NET相比，肺LCNEC具有特异的基因改变，包括*ADAMTS12*(20%)、*ADAMTS2*(15%)、*GAS7*(12%)以及*NTM*(10%); 其中，*GAS7*和*NTM*参与神经内分泌分化^[8]^。近年研究^[7, 12, 16]^还发现在肺LCNEC中存在*SMARCA2*、*NTRK2*、*NTRK3*基因突变。与SCLC相比，在肺LCNEC中*LAMA1*、*PCLO*及*MEGF8*的突变率明显增高，突变率分别是10%：2%、6%：1%和5%：0%(*P*值分别为0.019、0.023和0.015)^[11]^。

## 2 肺大细胞神经内分泌癌的分子分型

### 2.1 分子分型

目前，尚未对肺LCNEC进行精准的分子分型，原因在于相关研究不够充实。Rekhtman等^[7]^对45例肺LCNEC肿瘤组织及配对正常组织进行测序(next-generation sequence, NGS)，通过聚类分析(cluster analysis)把肺LCNEC分为3种分子亚型，一类是与SCLC相似(SCLC-like)的LCNEC，该类亚型的分子特点是共同出现*TP53*及*RB1*基因突变或缺失，该种基因突变较常出现于SCLC中; 同时还出现其他与SCLC一样的典型基因的改变，包括*MYCL*、*SOX2*、*FGFR1*扩增和*PTEN*突变或缺失; 第二类亚型是与非小细胞肺癌(non-small cell lung cancer, NSCLC)相似(NSCLC-like)的LCNEC，在该分型中没有*TP53*及*RB1*共同突变，但可观察到*STK11*、*KRAS*、*KEAP1*基因改变，而这些基因在NSCLC中较常出现; 第三类亚型基因特征是肿瘤突变负荷较前两种亚型低，并具有*MEN1*基因突变，该种基因特征的改变与肺TC相似。其后，George等^[8]^进一步分析了各亚型的特征：其中，I型LCNEC主要基因改变为*TP53*与*STK11/KEAP1*的共同突变或缺失，且表现为*ASCL1*及*DLL3*高表达和NOTCH通路失活; 而II型LCNEC主要基因改变是*TP53*及*RB1*突变或缺失，该型患者表现为*ASCL1*、*DLL3*的低表达及NOTCH通路的激活。

### 2.2 分子分型与临床治疗

近年有研究发现某些基因改变与化疗疗效相关。一项回顾性研究^[17]^对63例肺LCNEC患者的血液及组织进行基因测序，把存在*TP53*及*RB1*共同突变的患者定义为SCLC-like型，缺乏共同突变的定义为NSCLC-like型，并对54例患者的治疗及预后进行分析; 结果显示SCLC-like型LCNEC的患者较NSCLC-like型的患者预后差，但无统计学差异(9.8个月*vs* 14.4个月，*P*=0.18);且在SCLC-like LCNEC患者中，依托泊苷联合铂类的化疗方案与培美曲塞/吉西他滨/紫杉醇联合铂类方案相比，其客观缓解率(objective response rate, ORR)(75% *vs* 0%, *P*=0.02)及疾病控制率(disease control rate, DCR)(100% *vs* 20%, *P*=0.007)明显增高，明显延长患者无进展生存期(progression-free survival, PFS)(8.3个月*vs* 2.4个月，*P*=0.002);但患者总生存期(overall survival, OS)无明显改善(9.7个月*vs* 4.1个月，*P*=0.600);然而，在NSCLC-like LCNEC中，吉西他滨/紫杉醇联合铂类治疗的患者与依托泊苷或培美曲塞联合铂类方案治疗的患者相比，PFS(2.5个月*vs* 5.5个月，*P*=0.045)及OS(9.4个月*vs* 19.6个月，*P*=0.07)明显更差。另有一研究^[18]^表明，在*RB1*基因野生型的LCNEC患者中，与依托泊苷联合铂类的化疗方案相比，吉西他滨/紫杉醇联合铂类的化疗方案可明显延长患者的OS(9.6个月*vs* 5.8个月，*P*=0.026);但是，在*RB1*突变的LCNEC患者中，未观察到化疗方案之间疗效的差异。

## 3 大细胞神经内分泌癌的分子标志物研究

### 3.1 诊断相关的分子标志物

肺LCNEC组织学特征复杂，活检标本不足以支持诊断，通常需要手术大标本，通过形态学及内分泌标志物来证实^[19, 20]^。典型的形态学特征如器官样结构、栅栏及花环样排列、NSCLC细胞学特点、低核质比、大量丰富的核仁和大量的坏死^[21]^;临床上常用的神经内分泌标志物有嗜铬粒蛋白A(chromogranin A, CGA)、突触小泡蛋白(synaptophysin, SYN)和神经细胞黏附分子(neural cell adhesion molecule, NCAM-1/CD56)^[21]^，而以上特点导致LCNEC诊断困难。因此，近年来研究者聚焦于LCNEC诊断标志物的研究，以寻找特异性及敏感性高的分子标志物。Bari等^[22]^对8例SCLC和8例LCNEC的冰冻组织进行测序，发现CDX2(Caudal type homeobox 2)、VIL1(Villin 1)及BAI3(brain-specific angiogenesis inhibitor 3)在两种肿瘤中表达差异大; 并通过RT-PCR及免疫组化验证发现，当CDX2与VIL1结合用于诊断肺LCNEC时，其敏感性及特异性高达81%，而BAI3在诊断SCLC时的敏感性和特异性分别为89%和75%;因此，CDX2、VIL1及BAI3具有潜在的区分LCNEC与SCLC的能力。微管不稳定蛋白(Stathmin-1, STMN1)具有调控微管的作用，并在NSCLC中证实了与肿瘤恶性程度有关^[23-25]^;同时也发现了在HGNET中*STMN1*表达明显高于NSCLC，该基因有助于鉴别NSCLC与HGNET^[26]^。人牛膝鳞片同源物1(human achaete-scute homolog 1, hASH1)可以调节神经内分泌细胞的生长^[27]^;Ye等^[28]^对肺NET(肺TC、SCLC及LCNEC)、肺腺癌(lung adenocarcinoma)及肺鳞癌(squamous cell lung carcinoma)进行hASH1免疫组化实验，发现在HGENT中表达比例最高(LCNEC为72.7%，SCLC为79.2%)，所以hASH1可对HGENT进行临床诊断。Morise等^[29]^对60例SCLC及45例LCNEC组织进行肿瘤干细胞相关标志物表达情况的分析，发现SOX2及CD166在SCLC及LCNEC中表达具有明显的差异，*P*值分别为0.003和0.046，可通过这两种分子的表达情况区分SCLC和LCNEC。现已知*DLL3*是ASCL1的下游靶基因，并参与肺NET的神经内分泌分化^[30, 31]^，而在一项研究^[32]^中发现DLL3在LCNEC中的表达率为74%(70/94)，同时也发现在TP53野生型和TP53突变型的LCNEC中DLL3表达有差异，且DLL3的表达与ASLC1的表达相关，以上说明DLL3与LCNEC的分子亚型和神经内分泌谱相关，可能具有成为鉴别LCNEC分子亚型的潜力。

### 3.2 预后相关的分子标志物

在肺LCNEC中，无标准指南推荐最优的治疗方案。现临床常用方案有两种，一种为NSCLC常规化疗方案，如培美曲塞、吉西他滨联合含铂化疗方案; 另一种为SCLC常规化疗方案，如依托泊苷联合铂类的化疗方案; 但肺LCNEC的疗效较NSCLC及SCLC的疗效差^[5]^。因此需预后相关分子筛选治疗有效的患者。研究者对LCNEC患者的肿瘤干细胞分子标志物进行分析，发现ALDH1表达阳性患者的无复发生存期(recurrence-free survival, RFS)和OS较ALDH1阴性患者的短(5年RFS率：39% *vs* 67%，*P*=0.009;5年OS率：50% *vs* 79%，*P*=0.021);并且在多因素*Cox*回归分析中，ALDH1阳性表达是独立的预后危险因素^[29]^。YAP1是Hippo通路上的主要效应因子，通常在NSCLC中表达^[33]^;有学者^[34]^在30例肺LCNEC中分析了YAP1的表达情况，18/30(60%)的患者出现*YAP1*的缺失，同时发现*YAP1*的缺失与神经内分泌标志物表达情况相关，且通过生存分析证明YAP1阴性患者对化疗更敏感。近年研究表明免疫微环境对抗肿瘤起到至关重要的作用，有研究^[35]^对肺LCNEC患者的术前外周血中中性粒细胞/淋巴细胞比(neutrophil-lymphocyte ratio, NLR)进行分析，证实NLR是LCNEC患者总生存期的预后独立影响因素(*P*=0.011, HR=8.559, 95%CI: 1.783-80.230)，因此，NLR具有成为预后相关的分子标志物的潜力。另有学者^[36]^发现在肺LCNEC患者中普遍表现为淋巴细胞轻度减少，而在治疗前具有明显T细胞(T-cell repertoire, TCR)亚群改变和更高淋巴细胞计数的患者，其治疗效果更好并且生存期更长(441 d *vs* 157 d, *P*=0.019)，同时还证实了治疗3个月后患者的TCR恢复程度越高，预后越好(OS: 617 d *vs* 316 d, *P*=0.036)。E-cadherin是钙黏蛋白家族中的成员，在细胞黏连中起到重要作用^[37]^，但其黏连作用需与β-catenin结合形成E-cadherin-β-catenin复合物才能发挥作用^[38]^;而E-cadherin-β-catenin复合物的缺失可导致肿瘤的恶化^[39]^。Salon等^[40]^对102例肺NET(16例肺TC，8例肺AC，37例LCNEC，41例SCLC)进行E-cadherin及β-catenin免疫组化实验，发现E-cadherin及β-catenin的表达缺失在HGNEC中更普遍(*P* < 0.000, 1)，且E-cadherin及β-catenin与淋巴转移相关(*P*=0.000, 1和*P*=0.000, 5)，所以该复合物的形成与神经内分泌癌的预后及恶化有关。*Klotho*是一种抑癌基因，参与离子通道和生长因子水平的调节，增强对氧化应激的抵抗力^[41, 42]^。在Brominska等^[43]^的研究中，Klotho阳性的肺LCNEC患者与Klotho阴性的患者相比，患者的OS明显延长(*P*=0.015, HR=0.37, 95%CI: 0.17-0.86)。有研究^[44, 45]^证实PD-L1在肿瘤细胞(tumor cells, TC)和炎症细胞(inflammatory cells, IC)中的表达可能是预测疗效的标志物，但在LCNEC中尚不清楚。Arpin等^[46]^在法国的17家中心中收集了105例LCNEC患者的组织标本，经过重新复阅后，最终筛选出了68例LCNEC患者。通过分析PD-L1在这些标本中的表达情况，发现与SCLC和NSCLC相比，TC-/IC+是LCNEC最常见的PD-L1的表达模式，且在炎症细胞中PD-L1表达情况似乎是一个独立的预后因素。

## 4 总结及展望

根据目前研究，肺LCNEC中基因图谱的改变同时与SCLC、NSCLC及肺TC重合; 说明肺LCNEC具有较高的异质性。并且对比其他类型的肺癌，肺LCNEC具有特异性基因的改变; 而这些基因可成为肺LCNEC诊断相关分子标志物。根据基因图谱的改变，已对肺LCNEC进行大致的分子分型，但还需大量数据验证。但在肺LCNEC分子分型研究中证实了不同分子亚型的肺LCNEC对化疗方案的疗效不同，且预后也不相同。并且通过对比不同亚型肺LCNEC的基因改变，可以发现潜在的预后分子标志物。

同时，基因图谱及分子分型的研究使得研究者对肺LCNEC的分子特性及分子机制进一步了解，并促进肺LCNEC进入精准治疗时代，从而提高肺LCENC的疗效，延长患者的生存期。而诊断及预后分子标志物的研究则可以提高肺LCNEC临床诊断的准确性及有效的筛选出生存获益的患者; 但是否能运用于临床，还需深入研究。

目前，肿瘤治疗已经进入免疫治疗时代; 而有关肺LCNEC免疫相关研究非常少。在免疫机制中，有研究^[36]^发现肺LCNEC可以诱导T细胞的变化，表明抗原诱导的T细胞增殖可能是其致病机制。而另一研究^[47]^对106例肺LCNEC患者的外周血进行血小板和白细胞计数，并通过生存分析发现NLR和血小板/淋巴细胞比(platelet-to-lymphocyte ratio, PLR)是肺LCNEC患者的独立预后因素，这暗示着细胞免疫与肺LCNEC的进展有关。而在免疫治疗的疗效研究中，Oda等^[48]^报道了1例混合型肺LCNEC在多程治疗后接受纳武利尤单抗(Nivolumab)治疗后获得长期生存的病例。另有一项临床试验^[49]^，研究者入组晚期肺LCNEC共37例; 其中21例患者接受免疫抑制剂(immune check-point inhibitor, ICPi)治疗，并与晚期NSCLC的免疫治疗疗效进行比对; 结果显示肺LCNEC免疫治疗疗效与NSCLC的免疫治疗疗效相当，而具体的临床数据需要进一步的研究，以上均说明肺LCNEC的发生发展与免疫及免疫微环境密不可分，且对免疫治疗具有一定的疗效，但筛选出可在免疫治疗中受益的患者，还需进一步研究。因此，对肺LCNEC的免疫微环境进行分子分型及发现有效的免疫治疗相关的预后分子标志物是我们的下一步研究方向。
